# Vascular peroxidase 1 is independently associated with worse kidney function in patients with peripheral artery disease

**DOI:** 10.1007/s40620-020-00818-w

**Published:** 2020-08-19

**Authors:** Lavinia Costas, Carsten Thilo Herz, Clemens Höbaus, Renate Koppensteiner, Gerit-Holger Schernthaner

**Affiliations:** 1 Division of Angiology, Department of Medicine II, Medical University and General Hospital of Vienna, Währinger Gürtel 18–20, Vienna, 1090 Austria; 2 Division of Endocrinology and Metabolism, Department of Medicine III, Medical University and General Hospital of Vienna, Währinger Gürtel 18–20, Vienna, 1090 Austria

**Keywords:** Vascular peroxidase 1, Oxidative stress, Peripheral artery disease, Chronic kidney disease

## Abstract

**Background:**

Oxidative stress is involved in cardiovascular disease such as peripheral artery disease (PAD). Vascular Peroxidase 1 (VPO1), a novel heme-containing peroxidase mainly expressed in the cardiovascular system, aggravates oxidative stress. Evidence in humans is limited. Current work aims to measure VPO1 in patients suffering from PAD, and to evaluate the association of VPO1 with conventional markers of cardiovascular risk factors (CVRF), including the estimated glomerular filtration rate (eGFR) and albuminuria categories.

**Methods:**

This study is part of a longitudinal observational study. At baseline, 236 PAD-patients were included. VPO1 plasma levels (ng/mL) were measured by commercially available ELISA kits. A two-sided p level of < 0.05 was considered statistically significant.

**Results:**

In the cross-sectional analysis (n = 236), VPO1 associated with ageing (p = 0.035) as well as with eGFR and albuminuria category, the markers of chronic kidney disease (CKD)-progression (p = 0.042). The longitudinal 18-months follow-up analysis (n = 152) demonstrated that baseline VPO1 predicts rapid kidney function decline (RKFD) (n = 49), defined as more than − 3 mL/min/1.73m^2^ eGFR loss per year, (OR per one SD VPO1 1.60 (1.11–2.30); p = 0.009). This association between VPO1 and kidney function withstood the multivariable adjustment for traditional CVRF including baseline eGFR and urine albumin-to-creatinine ratio (UACR), (adjOR per one SD VPO1 1.73 (1.14–2.61); p = 0.046).

**Conclusion:**

This study is first to reveal that VPO1 is independently associated with declining kidney function in patients with PAD. VPO1 shows a tighter association to kidney function than to other CVRF. This finding points to VPO1 as a potential target protein to assess CKD-progression.

## Introduction

Peripheral artery disease (PAD) is one of the most common initial presentations of cardiovascular disease diagnosed in elderly patients with type 2 diabetes mellitus (T2DM) [1]. While a multitude of risk factors (such as T2DM) contribute to PAD [2], chronic kidney disease (CKD) has emerged as a significant independent cardiovascular risk factor (CVRF) [3]. In this context, kidney function is described as a determinant of endothelial dysfunction [4]. Oxidative stress is a pathophysiological factor for PAD progression [5] and related CVRF [6, 7].

Vascular peroxidase 1 (VPO1), also known as human peroxidasin homolog, is a newly identified member of the heme-containing peroxidase family [8]. VPO1 is primarily expressed in the vascular wall, both in endothelial cells and smooth muscle cells, and secreted into the blood stream [8]. This peroxidase aggravates oxidative stress through utilizing hydrogen peroxide to produce hypohalous acids [9].

Pathophysiologically, VPO1 promotes oxidation of low-density lipoprotein (LDL) and mediates formation of foam cells, a hallmark of atherogenesis [10]. Moreover, VPO1 plays a role in endothelial dysfunction present in rats with T2DM [11]. Hypohalous acid-derived modification of renal tissues, specifically collagen IV networks, contributes to functional protein damage in experimental diabetic nephropathy models [12]. Furthermore, the expression of peroxidasin (a synonym for VPO1) is increased in a mouse model of renal fibrosis induced by unilateral ligation of the ureter [13]. Anti-peroxidasin antibodies are present in pulmonary-renal syndromes, such as Goodpasture disease, ANCA-associated vasculitis and systemic lupus erythematosus [14]. Intriguingly, VPO1 is likely to carry out peroxidative reactions previously attributed exclusively to myeloperoxidase (MPO), the protagonist member of the heme-containing peroxidase family [8]. Peroxidasin but not MPO plays a role in tubulointerstitial fibrosis in the murine unilateral ureteral obstruction model [15]. This finding points to VPO1 as a potential target protein, as renal fibrosis represents a pathologic hallmark of CKD.

The routine clinical assessment of CKD is established by a decreased kidney function reflected through a decreased estimated glomerular filtration rate (eGFR), or by the presence of kidney damage inferred from clinical markers such as albuminuria [16]. While the cause of CKD is an important predictor of disease progression, it is the alteration in the categories of eGFR and albuminuria that are used to assess CKD-progression [16]. The ability to quantify changes is pivotal in providing mechanistic insights into disease pathophysiology.

While several studies have been performed on the role of VPO1 at the cellular level or in animal models [10, 11, 13], data on an association of VPO1 with clinical parameters of humans under atherosclerotic conditions are missing. Increased concentrations of VPO1 are consistent among pre-clinical studies addressing cardiovascular disorders, but it is currently unclear whether an elevated level of circulating VPO1 accompanies severity of commonly available clinical markers of CVRF, in humans.

This work is aimed at exploring the association of VPO1 and CVRF that are present in PAD, an occurrence of cardiovascular disease. The character of the study is not mechanistic, and hence it is not aimed at explaining the potential complex biological pathways underlying this association.

## Materials and methods

### Study collective

The current investigation is part of the prospective VMC (vascular medicine center) Vienna observational study of PAD-patients [17], and was designed as exploratory data analyses of longitudinally acquired data. Research participants were recruited at the Division of Angiology, Department of Medicine II of the Medical University and General Hospital of Vienna. The study included women and men between 40 and 90 years of age, with PAD Fontaine stage I or stage II, and excluded patients having type 1 diabetes mellitus, serum creatinine level above 3 mg/dL, hormone replacement therapy, connective tissue disease, malignant disease, or critical illness within the last six months [17]. Based on the eligibility criteria of the main study, a subset of 236 stable participants, who returned for the first follow-up visit at 6 months, were enrolled for the purpose of the current study.

### Analysis parameters

Diagnosis of PAD utilizing the Fontaine classification was confirmed by the ankle-brachial index (ABI value ≤ 0.9) or the toe-brachial index in patients with media-sclerosis (TBI value ≤ 0.7), respectively, following the recommendations of the Trans-Atlantic Inter-Society Consensus II for the Management of Peripheral Arterial Disease Working Group [18].

T2DM status was defined by the American Diabetes Association ‘Diagnosis and Classification of Diabetes Mellitus’ [19].

Smoking status was classified as current or former smoker versus never smoker. Respondents who had smoked at least 100 cigarettes in their lifetime were defined as former smokers.

Arterial hypertension was defined as blood pressure higher than or equal to 140 mmHg systolic over 90 mmHg diastolic in at least two measurements, or in case of current intake of anti-hypertensive drugs.

Kidney function was assessed by estimating the glomerular filtration rate from serum concentration of creatinine, using the CKD-EPI formula [16]. This study adhered to the KDIGO 2012 Clinical Practice Guideline for the Evaluation and Management of Chronic Kidney Disease [16]. Likewise, decreased eGFR below 60 mL/min/1.73m^2^ defined CKD, and urine albumin-to-creatinine ratio (UACR) from spot urine beyond 30 mg/g defined abnormally elevated UACR. In addition, patients were classified for CKD combining eGFR and albuminuria categories, ranging from CKD G1 A1 to CKD G4 A3. They were divided into 4 categories of risk of CKD-progression, ranging from low risk to very high risk [16].

Considering the longitudinal design of the underlying main study [17], an analysis of available follow-up data of eGFR after 18 months was performed, as an approach to test whether deterioration of kidney function over time associates with higher baseline VPO1 levels, among diabetic and non-diabetic PAD-patients. The reference value for eGFR slope is − 1.07 ± 0.42 mL/min/1.73m^2^ per year [20]. Rapid kidney function decline (RKFD) is defined as more than − 3 mL/min/1.73m^2^ per year, respectively more than − 4.5 mL/min/1.73m^2^ per 18 months [21, 22].

### Quantitative determination of VPO1

Venous blood samples were drawn after an overnight fast, centrifuged, and stored frozen at − 80 °C, avoiding loss of bioactivity and contamination. After an overnight thawing, concentrations of VPO1 (ng/mL) were measured in patients’ plasma by an enzyme-linked immunosorbent assay (ELISA), using commercially available human kits (Cusabio Biotech Co. Ltd, Wuhan, China), following the manufacturer’s instructions [23]. Values of samples below the quantification limit of 0.188 ng/mL were imputed with this value. Intra-assay and inter-assay coefficients of variation (CV) were less than 8% and 10%, respectively.

### Statistical analyses

Statistical methods were performed applying the statistical software package SPSS 23 (IBM SPSS Inc., Chicago, IL, USA). Normal distribution was confirmed using the Kolmogorov–Smirnov test. Quantitative variables with normal distribution (including VPO1) were displayed as mean ± standard deviation (SD), whereas continuous variables with non-parametric distribution were given as median with interquartile range (median; 25th percentile, 75th percentile). Categorial data, including nominal, ordinal and dichotomous variables, were represented by number (n) and percentage (%). In order to reduce skewness of distributions in statistical analyses, the variables UACR and C-reactive protein (CRP) were logarithmically transformed.

Statistical analyses compromised student’s unpaired t-test, Mann–Whitney U, analysis of variance (ANOVA), Kruskal–Wallis (K-W), paired sample t-test, Pearson’s Chi-square (χ^2^) test, Fisher’s exact test and binary logistic regression analyses, as appropriate. The cardiovascular-related confounders (age, gender, smoking status, HbA1_c_, systolic blood pressure (SBP), LDL, CRP as well as eGFR and UACR) were included as covariates in multivariable models. Adjusted odds ratios (adjOR) were determined by multivariable analyses and presented per one SD increase (95% confidence interval (CI)). Confounding was identified by a change in OR greater than 10.0%, according to Rothman [24]. Constructing a receiver operating characteristic (ROC) curve, the area under the curve (AUC) was computed. All statistical assessments were evaluated two-sided at a significance level of < 0.05; a two-tailed probability level of < 0.01 was considered highly significant.

## Results

The study enrolled 236 participants, more specifically 128 patients with PAD Fontaine stage I (69.1 ± 9.8 years; 64.1% male; 41.4% T2DM) and 108 patients with stage II (68.8 ± 11.0 years; 73.1% male; 53.7% T2DM), (all p ≥ 0.05), constituting the baseline population.

VPO1 mean levels did not differ significantly between persons without and with claudication symptoms (Fontaine stage I, 1.94 ± 0.83 ng/mL vs. Fontaine stage II, 2.06 ± 0.90 ng/mL; p = 0.263). VPO1 was evenly distributed between T2DM and without T2DM (2.02 ± 0.88 ng/mL, n = 111 vs. 1.97 ± 0.86 ng/mL, n = 125; p = 0.625).

### VPO1 and baseline characteristics

Baseline characteristics are shown across VPO1 tertiles, in Table 1. A monotone positive association was found with patient age (p = 0.035). Of all anthropometric, biochemical and clinical parameters assessed, renal parameters were associated with VPO1 concentrations (creatinine: p = 0.006, UACR: p = 0.030; eGFR: p = 0.004).

**Table 1:** Baseline characteristics across VPO1 tertiles

Baseline characteristics	n = 236	V P O 1 ng/mL			p value
		Tertile I	Tertile II	Tertile III	
		0.19–1.64	1.641–2.37	2.371–4.13	
		n = 78	n = 79	n = 79	
VPO1 ng/mL	2.00 ± 0.87	1.03 ± 0.43	1.99 ± 0.21	2.95 ± 0.43	
Age years	69.0 ± 10.3	67.2 ± 10.8	68.4 ± 10.8	71.3 ± 9.0	0.035*
Gender, male	161 (68.2%)	48 (61.5%)	54 (68.4%)	59 (74.7%)	0.209
BMI kg/m^2^	27.25 ± 4.01	26.93 ± 3.61	27.71 ± 4.21	27.10 ± 4.18	0.441
Smoking	191 (80.9%)	60 (76.9%)	69 (87.3%)	62 (78.5%)	0.201
ABI	0.74 ± 0.20	0.72 ± 0.19	0.75 ± 0.20	0.74 ± 0.22	0.619
TBI	0.50 ± 0.18	0.46 ± 0.14	0.51 ± 0.22	0.51 ± 0.17	0.644
Glucose mg/dL	103.0; 93.0, 125.3	104.0; 93.5, 126.5	102.0; 94.0, 124.0	103.0; 92.0, 124.5	0.906
HbA1_c_ rel. %	6.0; 5.7, 6.5	6.0; 5.7, 6.7	6.1; 5.7, 6.4	5.9; 5.6, 6.4	0.318
Hypertension	208 (88.1%)	65 (83.3%)	70 (88.6%)	73 (92.4%)	0.211
SBP mmHg	130; 120, 145	130; 120, 150	130; 120, 150	130; 120, 140	0.762
DBP mmHg	75; 70, 80	75; 70, 80	75; 70, 80	75; 65, 80	0.462
ACE-I or ARB	178 (75.4%)	57 (73.1%)	57 (72.2%)	64 (81.0%)	0.364
Hyperlipidemia	229 (97.0%)	76 (98.7%)	77 (97.5%)	76 (97.4%)	1.000
Triglyc mg/dL	131.50; 98.00, 190.25	121.00; 95.50, 161.00	144.00; 101.00, 202.00	133.50; 99.75, 213.50	0.062
Total Chol mg/dL	180.69 ± 36.06	174.41 ± 34.06	189.27 ± 39.23	178.13 ± 33.27	0.027*
LDL mg/dL	97.83 ± 30.44	95.94 ± 28.44	103.93 ± 33.86	93.51 ± 28.00	0.080
HDL mg/dL	52.43 ± 13.61	51.28 ± 13.29	54.32 ± 13.84	51.65 ± 13.66	0.315
Statins	206 (87.3%)	72 (92.3%)	66 (83.5%)	68 (86.1%)	0.238
Crea mg/dL	1.03; 0.92, 1.22	0.99; 0.92, 1.20	1.02; 0.92, 1.18	1.12; 0.98, 1.34	0.006**
eGFR mL/min/1.73m^2^	66.31 ± 18.08	70.01 ± 18.41	68.07 ± 15.52	60.86 ± 19.08	0.004**
UACR mg/g	10.00; 5.00, 33.00	6.00; 4.00, 27.00	7.00; 5.00, 30.75	14.50; 6.00, 57.00	0.030*
CRP mg/dL	0.26; 0.15, 0.54	0.30; 0.15, 0.60	0.27; 0.11, 0.51	0.24; 0.15, 0.46	0.440

VPO1 concentrations were significantly associated with renal parameters

Statistical analyses included analysis of variance, Kruskal–Wallis, Pearson’s Chi-square test and Fisher’s exact test, as appropriate.

Data presented as mean ± standard deviation or median; 25th percentile, 75th percentile or n (%), as appropriate.

^*^Indicates statistical significance, p < 0.05.

**Indicates high statistical significance, p < 0.01.

*ACE-I* angiotensin-converting-enzyme inhibitor, *ABI* ankle-brachial index, *ARB* angiotensin-II-receptor blocker, *BMI* body mass index, *Crea* creatinine, *CRP* C-reactive protein, *DBP* diastolic blood pressure, *eGFR* estimated glomerular filtration rate, *HbA1*_*c*_ glycated hemoglobin A1_c_, *HDL* high-density lipoprotein, *LDL* low-density lipoprotein, *SBP* systolic blood pressure, *TBI* toe-brachial index, *Total Chol* total cholesterol, *Triglyc* triglyceride, *UACR* urine albumin-to-creatinine ratio

### VPO1 and chronic kidney disease

**Fig. 1:**
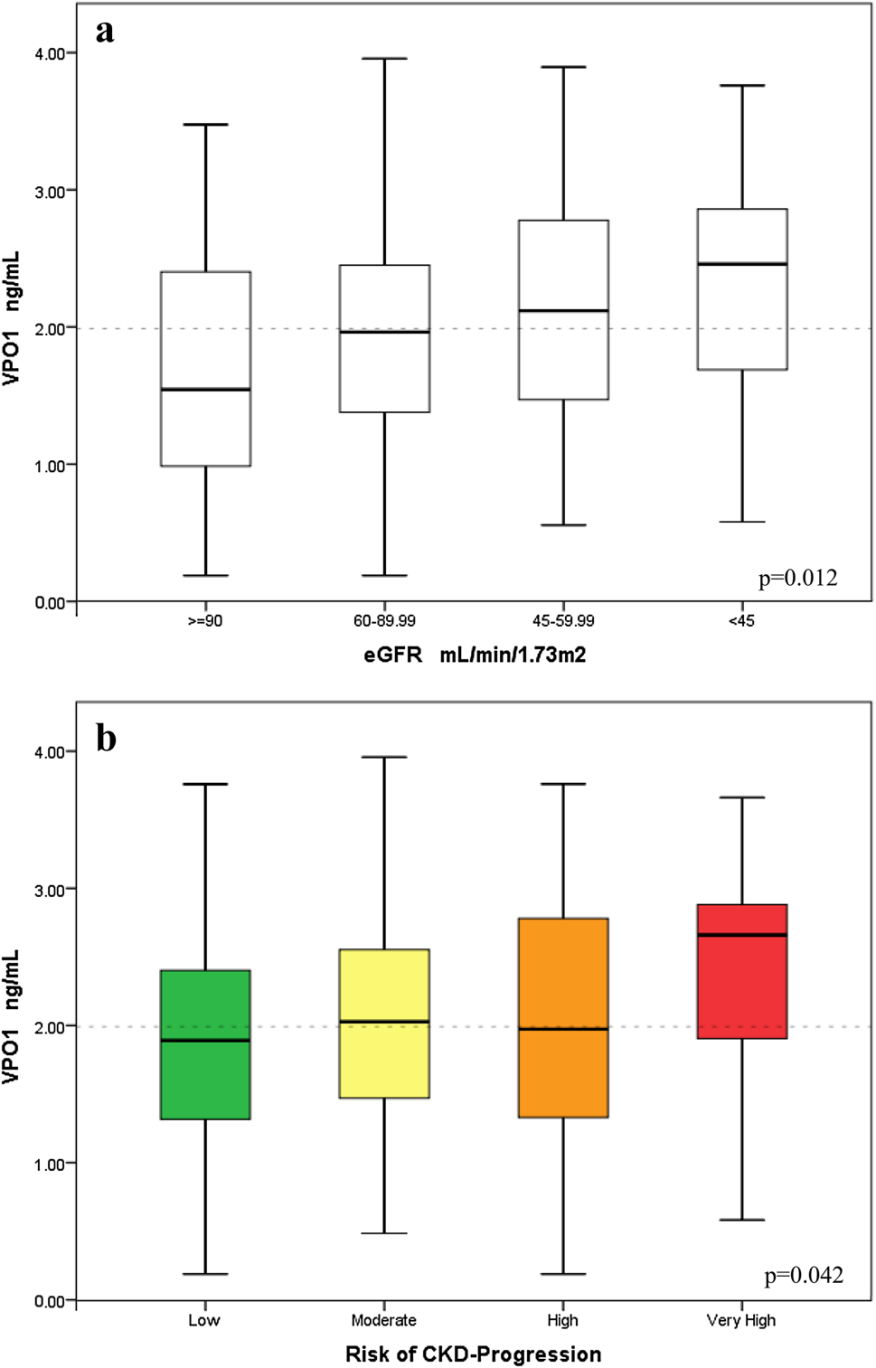
**a** VPO1 and kidney function. Decreasing kidney function was significantly associated with an increasing VPO1 level. Statistical analysis included analysis of variance. ≥ 90 mL/min/1.73m^2^, G1 (n = 24); 60–89.99 mL/min/1.73m^2^, G2 (n = 130); 45–59.99 mL/min/1.73m^2^, G3A (n = 48); < 45 mL/min/1.73m^2^, G3B–G4 (n = 32). *eGFR* estimated glomerular filtration rate. **b** VPO1 and risk of CKD-progression. VPO1 associated with higher risk of CKD-progression. Statistical analysis included analysis of variance. Low (n = 119), moderate (n = 50), high (n = 32), very high (n = 23). *CKD* chronic kidney disease

Decreasing kidney function estimated by eGFR category (G1–G4) was associated with an increasing VPO1 level (p = 0.012), as displayed in Fig. 1a. In addition, VPO1 increased with albuminuria group (A1–A3) from those with normal to mildly (UACR < 30 mg/g), moderately (UACR 30–300 mg/g), and severely (UACR ≥ 300 mg/g) increased albuminuria (p = 0.047). Further, VPO1 was analyzed in relation to clinical risk assessment of CKD-progression, including eGFR and albuminuria categories simultaneously [16]. Fig. 1b outlines the increased VPO1 in patients at higher risk of CKD-progression (p = 0.042).

Since univariate analyses suggested an association between VPO1 and renal parameters, binary logistic regression analyses on dichotomized kidney function, at the eGFR cut-off point of 60 mL/min/1.73m^2^, were performed, intended to examine the impact of other CVRF. Firstly, simple binary logistic regression analysis was calculated taking continuous VPO1 values as independent variable (OR VPO1 1.45 (1.09–1.93); p = 0.010). No confounding was noted between the simple model and the adjusted model for gender, smoking status, HbA1_c_, SBP, LDL, logCRP and logUACR (adjOR VPO1 1.40 (1.02–1.92); p < 0.001). Additional adjusting for medication including ACE-I or ARB and statins, did not affect aforementioned model (adjOR VPO1 1.40 (1.01–1.93); p < 0.001). According to the adjusted pseudo-R^2^ measure Nagelkerke, 24.8% of the alteration in eGFR status was explained by this multivariable model; the AUC was computed at 0.756.

### VPO1 and kidney function decline

In order to explore this observation further, longitudinal analyses investigating kidney function decline were assessed. Follow-up data were available 18 months after baseline for 152 female and male adult participants. Patients in this subgroup were similar to the whole study population in regard to PAD Fontaine stage, T2DM status and in any baseline characteristic (all p ≥ 0.05), with the exception from a decreased LDL level (94.82 ± 28.60 vs. 103.20 ± 32.98 mg/dL; p = 0.043).

During the follow-up period of 18 months, average rate of change in eGFR ran up to − 1.63 ± 7.67 mL/min/1.73m^2^ (p = 0.010), resulting in a change of − 1.09 ± 5.11 mL/min/1.73m^2^ per year. The deterioration in renal function over time was more pronounced in patients within higher VPO1 tertiles at baseline (tertile I (0.59 ± 4.55 mL/min/1.73m^2^ per year), tertile II (− 1.43 ± 4.81 mL/min/1.73m^2^ per year) and tertile III (− 2.02 ± 5.57 mL/min/1.73m^2^ per year), (p = 0.036)). RKFD (> − 4.5 mL/min/1.73m^2^ per 18 months) was present in 49 patients (32.2%). RKFD was present in 41.2% of patients with T2DM in comparison to 25.0% without T2DM, (p = 0.034). Baseline eGFR mean levels were similar in those with RKFD and those without RKFD (66.11 ± 15.61 vs. 66.05 ± 18.29 mL/min/1.73m^2^; p = 0.984). Follow-up eGFR levels were lower (55.73 ± 15.12 vs. 68.58 ± 19.00 mL/min/1.73 m^2^; p < 0.001).

Patients with a RKFD exhibited significantly higher VPO1 as well as elevated UACR at baseline. Both VPO1 (p = 0.009) and logUACR (p = 0.004) revealed to be individually associated with higher risk of RKFD over time (OR VPO1 1.60 (1.11–2.30); OR logUACR 1.67 (1.17–2.38)). In the multivariable binary logistic regression model, elevated VPO1 and logUACR were associated with RKFD (p = 0.046), after correction for the confounders age, gender, smoking status, HbA1_c_, SBP, LDL, logCRP and baseline eGFR (adjOR VPO1 1.73 (1.14–2.61); adjOR logUACR 1.70 (1.15–2.51)). In order to assess the influence of the associated renal parameters as well as their impact on the proposed model, it was re-analyzed excluding the covariates eGFR and logUACR (adjOR VPO1 1.76 (1.19–2.60)). Subsequent adjustment for excluded renal parameters did not alter the initial model. This indicated that the prognostic power of VPO1 is independent from renal parameters.

Additionally, two models predicting RKFD were compared, one including only traditional CVRF, the other adding the biomarker VPO1. Table 2 depicts that inclusion of eGFR and logUACR (Model 1) improved RKFD risk discrimination beyond CVRF enumerated in the basic model, as the AUC increased by 0.068. A further improvement was evident when VPO1 was added (Model 2).

**Table 2:** Multivariable binary logistic regression models for rapid kidney function decline

Rapid kidney Function decline Basic model	Age years Gender Smoking HbA1_c_ rel. % SBP mmHg LDL mg/dL logCRP mg/dL	Adjusted R^2^ = 03.2% AUC = 0.602
Model 1	+ eGFR mL/min/1.73m^2^ + logUACR mg/g	Adjusted R^2^ = 09.9% AUC = 0.670
Model 2	+ eGFR mL/min/1.73m^2^ + logUACR mg/g + VPO1 ng/mL	Adjusted R^2^ = 15.6% AUC = 0.722

Inclusion of renal parameters (model 1), as well as of VPO1 (model 2) improved rapid decline risk discrimination beyond parameters enumerated in the basic model.

Statistical analyses included the multivariable binary logistic regression analyses and the adjusted pseudo-R^2^ measure Nagelkerke; constructing a ROC curve, the area under the curve was computed.

*AUC* area under the curve, *eGFR* estimated glomerular filtration rate, *HbA1*_*c*_ glycated hemoglobin A1_c_, *LDL* low-density lipoprotein, *logCRP* logarithmised C-reactive protein, *logUACR* logarithmised urine albumin-to-creatinine ratio, *R*^*2*^ coefficient of determination, *ROC* receiver operating characteristic, *SBP* systolic blood pressure

**Fig. 2:**
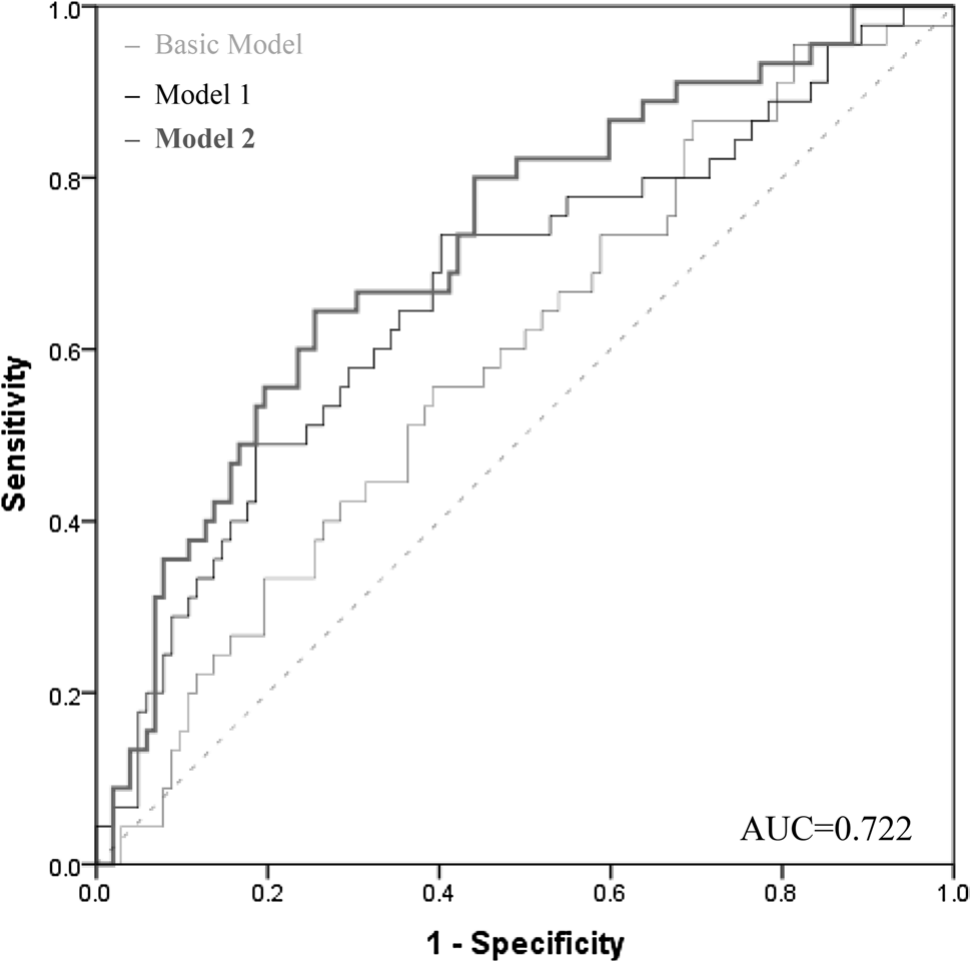
ROC curves for rapid kidney function decline. VPO1 added a benefit to rapid decline risk discrimination on top of traditional markers. Constructing a ROC curve, the area under the curve was computed. Basic model: adjusted for age, gender, smoking status, HbA1_c_, SBP, LDL, logCRP. Model 1: adjusted for age, gender, smoking status, HbA1_c_, SBP, LDL, logCRP, as well as eGFR and logUACR. Model 2: adjusted for age, gender, smoking status, HbA1_c_, SBP, LDL, logCRP, as well as eGFR, logUACR and VPO1. *AUC* area under the curve, *eGFR* estimated glomerular filtration rate, *HbA1*_*c*_ glycated hemoglobin A1_c_, *LDL* low-density lipoprotein, *logCRP* logarithmised C-reactive protein, *logUACR* logarithmised urine albumin-to-creatinine ratio, *ROC* receiver operating characteristic, *SBP* systolic blood pressure

The explained variability of RKFD improved, as R^2^ increased to 15.6%, as well as the predictive performance of RKFD improved, as the calculated AUC value increased further by 0.052 (p = 0.091, calculated with MedCalc Statistical Software). The ROC curve in Fig. 2 illustrates the added benefit of VPO1 to risk discrimination modeled on top of traditional CVRF.

## Discussion

We were first to explore VPO1 related to CVRF that are present in patients with PAD, revealing a correlation between VPO1 and worsening kidney function. VPO1 levels associated with eGFR as well as with changes in eGFR, confirming its association with kidney function.

Individuals displaying RKFD exhibited higher baseline VPO1 levels compared to those without. The contribution of UACR as well as of VPO1 to risk prediction was greater than that of any other CVRF. Noteworthy, the prognostic value of VPO1 was independent of UACR, the well-known predictor of kidney function decline [16]. Our statistical investigations suggest that VPO1 improved risk discrimination on top of the traditional clinical CVRF, including patient age, gender, smoking status, HbA1_c_, SBP, LDL, CRP as well as baseline eGFR.

Our results are in accordance with previous experimental pre-clinical studies [12, 13, 15]. Moreover, our findings are in line with pathophysiological concepts described in similar designed studies addressing other biomarkers. The reliable oxidative stress marker 8-isoprostane increases with advanced progression of CKD stage and correlates inversely with eGFR values [25]. The biomarker 5-Methoxytryptophan is described to attenuate renal fibrosis in mouse kidneys after unilateral ureteral obstruction, and its level decreases with CKD-progression [26]. The tubular damage marker retinol-binding protein 4 acts as an independent predictor of decreased kidney function [27]. A panel of biomarkers representing different pathways of kidney disease progression, including endothelial dysfunction and fibrosis, was shown to improve prediction of RKFD on top of traditional risk markers [21]. A combination of markers of tubular renal impairment and traditional risk parameters are reported to have a higher sensitivity and specificity on predicting kidney function decline than albuminuria alone [27].

At this point in time, to the best of our knowledge, we have not observed any study exploring VPO1’s relation to the cardiovascular system, in a PAD cohort. Our study revealed that VPO1 mean levels did not differ between the PAD stages addressing asymptomatic and symptomatic severity grades. Similarly, a study performed in 156 PAD-patients detected no correlation between MPO levels and ABI values [28]. Evidence linking VPO1 with atherosclerosis in humans is limited, with shortcomings in control for risk factors such as T2DM; nevertheless a previous study performed by Si-Yu Liu et al. has considered VPO1 related to endothelial dysfunction and inflammation in diabetes [11]. However, mentioned investigation reporting upregulated VPO1 expression in cultured endothelial cells pre-treated with high glucose, did not address the reference range of glucose values relevant to clinical scenarios. Our study performed in 236 PAD-patients failed to assess a difference in VPO1 mean levels between diabetics and non-diabetics.

This study has its limitations. The protein VPO1 represents a relatively unexplored research area, therefore, generated study hypotheses have to be interpreted carefully. VPO1 is not yet deemed to serve as a biomarker in clinical decision making. In order to determine whether VPO1 is actually a non-invasive marker, mediator and predictor of disease, as well as to determine to what extent assessment of VPO1 might contribute to risk profiling in patients with PAD, further prospective studies are necessary to elucidate associated comorbidities, as PAD as well as CKD have multifactorial etiologies. Due to current study design we cannot infer that the association between VPO1 and kidney function is causal. Another potential limitation is that this single-center study was performed in a Caucasian patient cohort. Thus, generalization of these findings should be validated in other patient cohorts.

Individuals with RKFD are reported to have increased cardiovascular as well as all-cause mortality risks, regardless of baseline demographic and kidney function parameters [22]. Our Cox proportional hazard model detected no association concerning VPO1 and 5-years all-cause mortality risk (p = 0.440; data not shown). However, we investigated VPO1 in a homogenous stable group of diseases; patients with more severe PAD Fontaine stage III and IV as well as patients with kidney failure were not included.

The protein VPO1 is promising to reflect kidney function in PAD Fontaine stage I and stage II, independent of UACR and other cardiovascular-related risk parameters.
